# Feeling the World Differently: Sensory and Emotional Profiles in Preschool Neurodevelopmental Disorders

**DOI:** 10.3390/children12070958

**Published:** 2025-07-21

**Authors:** Federica Gigliotti, Maria Eugenia Martelli, Federica Giovannone, Carla Sogos

**Affiliations:** Department of Human Neuroscience, Sapienza University of Rome, 00185 Rome, Italy; federica.gig@uniroma1.it (F.G.); mariaeugenia.martelli@uniroma1.it (M.E.M.); federica.giovannone@uniroma1.it (F.G.)

**Keywords:** neurodevelopmental disorders, preschool children, sensory processing, emotional–behavioral functioning, autism spectrum disorder, transdiagnostic approach, latent profile analysis

## Abstract

**Highlights:**

**What are the main findings?**

- Three distinct sensory profiles were identified in preschoolers with neurodevelopmental disorders, transcending diagnostic categories.
- Sensory profiles were closely associated with emotional–behavioral functioning, independently of diagnosis.

**What is the implication of the main finding?**

- Early sensory profiling may help detect emotional vulnerability in young children with neurodevelopmental conditions.
- Sensory assessments can support a dimensional and personalized approach to early intervention planning.

**Abstract:**

Background/Objectives: Atypical sensory processing is increasingly recognized as a transdiagnostic dimension of neurodevelopmental disorders (NDDs), with critical implications for emotional and behavioral regulation. This study aimed to identify distinct sensory profiles in preschool children with NDDs and to examine their associations with emotional–behavioral and cognitive/developmental functioning. Methods: A total of 263 children (aged 21–71 months) diagnosed with autism spectrum disorder (ASD), language disorder (LD), or other NDDs (ONDD) were recruited. Sensory processing was assessed using the SPM-P, emotional–behavioral functioning was assessed via the CBCL 1½–5, and cognitive/developmental levels were assessed through standardized instruments. Latent profile analysis (LPA) was conducted to identify sensory subtypes. Group comparisons and multinomial logistic regression were used to examine profile characteristics and predictors of profile membership. Results: Three sensory profiles emerged: (1) Multisystemic Sensory Dysfunction (20.1%), characterized by pervasive sensory and emotional difficulties, primarily observed in ASD; (2) Typical Sensory Processing (44.9%), showing normative sensory and emotional functioning, predominantly LD; and (3) Mixed Subclinical Sensory Processing (35%), with subclinical-range scores across multiple sensory and emotional domains, spanning all diagnoses. Higher cognitive functioning and fewer internalizing symptoms significantly predicted membership in the typical profile. A gradient of symptom severity was observed across profiles, with the Multisystemic group showing the most pronounced emotional–behavioral impairments. Conclusions: Distinct sensory–emotional phenotypes were identified across diagnostic categories, supporting a dimensional model of neurodevelopment. Sensory profiles were strongly associated with emotional functioning, independently of diagnostic status. Early sensory assessment may therefore offer clinically meaningful insights into emotional vulnerability and inform targeted interventions in preschool populations with NDDs.

## 1. Introduction

Over the past decade, there has been a growing recognition that sensory processing is not a peripheral or accessory dimension of neurodevelopmental disorders (NDDs), but rather a fundamental organizing principle of early brain development, shaping affective, cognitive, and behavioral trajectories from the earliest stages of life [1,2]. Sensory experiences represent the primary interface between the developing organism and its environment; thus, alterations in how sensory information is perceived, filtered, and integrated may have cascading effects on emotional regulation, learning, and social adaptation [3,4].

Many theoretical models have investigated individual differences in sensory processing modulation, identifying three atypical stimulus response modalities: hyperreactivity, hyporeactivity and sensory seeking [5,6]. Sensory hyperreactivity refers to an excessive response compared to the intensity of the perceived stimulus, which can be overstimulating and disturbing [5]. Conversely, hyporeactivity implies attenuated or absent responses to sensory stimuli, representing, sometimes, a risk condition, when lack of responses could compromise safety or social participation [7,8]. Sensory seeking refers to a marked, persistent and often atypical interest in sensory stimuli, which may trigger repetitive behaviors aimed to receive stimulations per se [9]. In autism spectrum disorder (ASD), atypical sensory behavior, including sensory-seeking, and repetitive behaviors are now recognized as a diagnostic feature according to DSM-5-TR [10].

Although atypical sensory processing has traditionally been framed within the diagnostic boundaries of autism spectrum disorder, current evidence suggests that sensory processing alterations are widely distributed across the neurodevelopmental spectrum, including attention-deficit/hyperactivity disorder (ADHD), intellectual disability (ID), and even early-onset affective disorders [2,11]. Individuals with ADHD, for example, often show atypical sensory seeking and poor registration, which may contribute to distractibility and impulsivity [12]. In contrast, sensory over-responsivity is more common in autism spectrum disorder and it is linked to maladaptive behavior, irritability and caregiver distress [13]. Sensory processing can be considered a cross-cutting neurobiological dimension and interacts dynamically with systems involved in affective and behavioral regulation across developmental stages, without necessarily constituting a symptom of any single health disorder [14].

From this perspective, sensory hyperreactivity, hyporeactivity, and sensory seeking can be understood not simply as behavioral epiphenomena, but as expressions of more fundamental regulatory dysfunctions. A child who reacts with panic to auditory input or who compulsively seeks tactile stimulation is not merely displaying odd behaviors, but may be struggling to achieve homeostatic regulation in the face of internal chaos or neurological noise [6,15].

The ability to modulate sensory input is deeply intertwined with the capacity for emotional self-regulation, attention allocation, and executive functioning [2,16]—core domains that are frequently compromised in NDDs [16,17].

Indeed, several studies have shown structural and functional alterations in central and supramodal brain areas, as well as in circuit connectivity, in NDDs. According to Cardon (2018), individuals with ADHD and ASD frequently exhibit abnormalities in the cerebellum and basal ganglia, as well as in higher-order functions, such as top-down regulation. These neurobiological alterations can influence sensory responsivity and, consequently, have a significant impact on neural plasticity and overall brain development [18]. Furthermore, mounting evidence suggests that sensory reactivity may not only contribute to emotional dysregulation but may actively shape its developmental trajectory. Children with heightened sensory sensitivity often show greater risk for anxiety and irritability [19], while those with under-responsivity may be misperceived as disengaged or oppositional, contributing to maladaptive interpersonal dynamics and increasing caregiver stress [11,20]. These patterns can be mutually reinforcing, creating developmental feedback loops in which sensory and emotional dysregulation compound over time. Emotional regulation, indeed, plays a crucial role in development and its trajectories. Difficulties in this domain may manifest as both externalizing and internalizing symptoms, such as anxiety, depression, conduct problems, and disruptive behaviors [2,14,19]. These symptoms are common in NDDs, particularly in ASD, and have a significant impact on quality of life. Children with neurodevelopmental disorders often exhibit maladaptive strategies, social withdrawal, reduced inhibitory control, and a more limited emotional vocabulary, which may be closely related to difficulties in regulating emotions [2,14].

Critically, most clinical models still treat sensory processing abnormalities as secondary features, often relegated to footnotes in diagnostic formulations or intervention plans. However, an emerging transdiagnostic perspective—consistent with frameworks such as RDoC (Research Domain Criteria)—invites us to reframe sensory processing as a domain of primary importance, potentially mediating the relationship between neural circuit dysfunction and behavioral phenotype across multiple disorders [1,21]. Such a perspective encourages clinicians and researchers to adopt dimensional approaches, capable of capturing the nuanced interplay between sensory, emotional, and behavioral functioning in children with complex neurodevelopmental profiles.

Grounded in a transdiagnostic and person-centered framework, the present study aimed to explore the heterogeneity of sensory processing in preschool-aged children with neurodevelopmental disorders (NDDs) and its associations with emotional and cognitive functioning. While most previous studies have focused on diagnostic group comparisons, we adopted a data-driven approach using latent profile analysis (LPA) to identify distinct sensory subtypes across a mixed clinical sample. This methodology allows for the detection of meaningful individual patterns that transcend diagnostic categories and may reflect early-emerging vulnerability profiles [22]. To our knowledge, few studies have applied LPA to characterize sensory profiles in preschoolers, and even fewer have examined their links with both emotional dysregulation and cognitive functioning. Specifically, we addressed the following research questions:

1. Is it possible to distinguish different transdiagnostic sensory profiles based on SPM-P scores in preschool-aged children with neurodevelopmental disorders?
2. Are there associations between sensory profiles and emotional dysregulation (e.g., internalizing problems), cognitive functioning, and age?
3. Do cognitive functioning and age contribute to determining sensory profiles, and if so, in what way?

## 2. Materials and Methods

### 2.1. Design and Participants

In this cross-sectional study, a total of 263 preschool-aged children (M:F = 4:1) with NDDs were included. Participants with NDDs were recruited from the Department of Human Neuroscience of Sapienza University of Rome—Outpatient Service for Neurodevelopmental Disorders between 2021 and 2024. These children ranged in age between 21 and 71 months. The total sample was divided into three main clinical groups depending on the diagnosed NDD: 73 patients with language disorder (LD) (age range = 29–67 months), 115 children with ASD (age range = 21–71 months), and 75 subjects with other neurodevelopmental disorder (ONDD) (age range = 23–71 months). For each group, age and gender ratio were statistically equivalent (χ^2^_(2)_ = 0.919, *p* = 0.632; χ^2^_(2)_ = 0.250, *p* = 0.882). Demographic information for the total sample and each diagnostic group is reported in Table 1.

All participants were diagnosed following a comprehensive neuropsychiatric evaluation which included a detailed medical history, clinical observation, and the use of standardized instruments.

For this study, specific inclusion and exclusion criteria were applied to ensure its validity. The inclusion criteria were as follows: (a) diagnosis of NDD, according to DSM-5-TR criteria; (b) age between 18 and 72 months; (c) no use of medications or other therapies. The exclusion criteria consisted of (a) history of associated neurological conditions, such as epilepsy or cerebral palsy; (b) known genetic syndromes; (c) severe sensory impairments; (d) history of traumatic brain injury; (e) inability to complete the assessment due to severe behavioral issues in the child or a lack of proficiency in the Italian language by the parents; (f) lack of informed consent.

Participation in this study was voluntary. Ethical approval was not required, as the assessments analyzed were conducted as part of routine clinical practice at our center, with no experimental procedures involved. Data were collected retrospectively in anonymized form, and written informed consent was obtained from the parents of all participants included. The study protocol adhered to the ethical standards outlined in the Declaration of Helsinki.

### 2.2. Measures

#### 2.2.1. Cognitive and Global Developmental Levels

Based on the child’s age, expressive language abilities, and capacity to interact and cooperate with the examiner, different standardized scales were administered to assess cognitive and global developmental levels.

The Griffiths Scales of Child Development—3rd Edition (Griffiths-III) is a tool designed to allow a detailed examination of developmental milestones in children aged 0–72 months. This instrument is composed of five scales: Foundations of Learning, assessing early problem-solving, reasoning, and concept acquisition; Language and Communication, evaluating expressive and receptive language skills; Eye and Hand Coordination, assessing fine motor abilities and coordination; Personal–Social–Emotional, evaluating emotion regulation, social interaction, and independence; and Gross Motor, assessing balance, postural control, locomotor skills, and gross body coordination. Furthermore, Griffiths-III provides a general development index (General Developmental Quotient—GDQ) that reflects the child’s overall developmental functioning [23,24].

The Leiter International Performance Scale—Revised (Leiter-R) is a nonverbal intelligence assessment tool specifically designed to measure cognitive abilities of individuals aged 2 to 20 years. This instrument does not require verbal instructions or responses, making it particularly effective for those who may face challenges with verbal communication. The Leiter-R consists of two batteries of ten subtests each: Visualization and Reasoning Battery (VR), assessing fluid reasoning, visualization skills, and problem-solving abilities, and Attention and Memory Battery (AM), evaluating sustained attention, working memory, and the ability to process and retain information. Intelligence Quotient (IQ) scores, reflecting the subject’s general nonverbal intellectual ability, are estimated based on the results of the VR battery subtests [25].

The Wechsler Preschool and Primary Scale of Intelligence—3rd Edition (WPSSI-III) is an assessment tool designed to evaluate the cognitive abilities and intellectual functioning of children ranging in age from 2 years and 6 months to 7 years and 3 months. This scale is organized into two age bands (2 years and 6 months to 3 years and 11 months; 4 years to 7 years and 3 months) to account for developmental differences in young children. The WPPSI-III provides multiple composite scores depending on the child’s age: Verbal Quotient, reflecting verbal reasoning, comprehension, and language-based skills; Performance Quotient, measuring nonverbal reasoning and problem-solving skills; Processing Speed Quotient, assessing the speed and accuracy of visual–motor coordination (for the older age band only); General Language Composite, an optional score that assess expressive and receptive language skills; and Full-Scale Intelligence Quotient, providing an overall estimate of child’s intellectual functioning [26,27]. All three scales are standardized by chronological age and are based on the same scoring system, with a mean score of 100 and a standard deviation (SD) of 15. In this study we considered the IQ for the Leiter-R and WPSSI-III scales and the GDQ for the Griffiths-III as indicators of general and cognitive developmental levels. In the ASD group, low functioning was defined by an IQ/DQ < 70.

#### 2.2.2. Autism Diagnostic Observation Schedule—2nd Edition (ADOS-2)

The ADOS-2 is a semi-structured, standardized diagnostic tool used for the assessment of individuals suspected of having ASD across their lifespan, from toddlers to adults. This instrument is designed to observe and measure a person’s communication (both verbal and nonverbal), social reciprocity, play, imaginative use of materials, and repetitive behaviors or restricted interests. The ADOS-2 comprises five different modules designed to assess individuals across different age groups and language abilities: Module Toddler, for young children ranging in age between 12 and 30 months who are nonverbal or do not yet produce phrase speech; Module 1, for children aged 31 months or older who do not consistently use phrase speech; Module 2, for individuals of any age who use phrase speech but are not yet fully verbally fluent; Module 3, for children and young adolescents, typically between the ages of 4 and 15 years, who have developed more advanced language skills for meaningful conversation; and Module 4, for adolescents and adults, typically aged 16 years or older, with advanced language skills who are capable of engaging in conversational dialog. The administration of the ADOS-2 takes approximately 30 to 60 min to complete and produces a diagnostic algorithm by scoring specific behaviors observed during the assessment. This tool generates Calibrated Severity Scores (CSS) for the domains of social affect and restricted and repetitive behaviors, in addition to a total score [28].

For the purpose of this study, we used Module Toddler, Module 1 or Module 2. Autistic symptom severity was quantified using the ADOS-2 total CSS, which ranges from 1 to 10. To enable direct comparisons with other ADOS-2 modules, the Toddler Module CSS was derived according to the methodology of Esler et al. (2015) [29].

#### 2.2.3. Sensory Processing Measure—Preschool (SPM-P)

The SPM-P is a standardized assessment tool specifically tailored to identify sensory processing challenges and highlight potential sensory integration dysfunctions that may affect daily functioning and behavior across different environmental contexts in children aged 2 to 5 years. This instrument consists of two primary rating forms, each comprising 75 items: Home Form, completed by parents or caregivers to assess the child’s sensory-related behaviors and social participation within the home environment, and School Form, completed by teachers or other school staff to evaluate the child’s sensory processing in the classroom and other school settings. Caregivers and teachers rate the frequency and intensity of specific behaviors using a 4-point Likert scale (1 = Never; 2 = Occasionally; 3 = Frequently; 4 = Always). Each form measures the child’s sensory processing across several domains: social participation (SOC), assessing the ability to engage with peers and adults in social contexts; vision (VIS), evaluating the child’s interpretation of and response to visual stimuli; hearing (HEA), addressing how the child processes and reacts to auditory input; touch (TOU), focusing on responses to tactile stimuli and textures; body awareness (BOD), which measures perception of body position and movement; balance and motion (BAL), assessing the ability to manage balance, spatial orientation, and movement; and planning and ideas (PLA), evaluating the ability to conceive and execute novel motor actions or responses. Furthermore, this tool provides a total sensory system score (TOT) derived from sensory processing subscales (VIS, HEA, TOU, BOD, BAL) plus several items related to taste and smell (TAS). Raw scores for each SPM-P scale are converted into T-scores using normative data, except for TAS subscale, for which they are not available. Within each sensory system, the SPM-P also identifies specific processing vulnerabilities: under-responsiveness, sensory-seeking behavior, over-responsiveness, ideation, postural control, motor planning, and perception [30,31].

For the purpose of this study, the Italian version of the Home Form was administered to the parents of all participants.

#### 2.2.4. Child Behavior Checklist for Ages 1½-5 Years (CBCL 1½-5)

The CBCL 1½-5 is a standardized and comprehensive assessment instrument designed to evaluate a wide range of emotional, behavioral, and social functioning issues in children aged 18 months to 5 years. This tool comprises 99 items rated by parents or primary caregivers on a 3-point Likert scale, ranging from 0 (no behavior) to 2 (behavior present frequently), based on the presence and frequency of specific behaviors. The CBCL 1½-5 provides measures of seven syndrome scales (emotionally reactive, anxious/depressed, somatic complaints, withdrawn, sleep problems, attention problems and aggressive behavior) and five DSM-oriented scales (Affective Problems, Anxiety Problems, Pervasive Developmental Problems, Attention Deficit/Hyperactive Problems and Oppositional Defiant Problems). Furthermore, the CBCL 1½-5 aggregates results into three broader domains (Internalizing Problems, Externalizing Problems, and Total Problems). Raw scores for each scale are converted into T-scores using normative data from age- and gender-matched peers [32].

In this study, we adopted the Italian version of this parent-report questionnaire, focusing on the T-scores of the DSM-oriented scales and the broad-band scales [33].

### 2.3. Statistical Analysis

All statistical analyses were performed using jamovi software (version 2.5.5; The jamovi project, 2023). The normality of continuous variables was assessed through the Shapiro–Wilk test, as well as visual inspection of Q-Q plots and histograms. Descriptive statistics were computed for sociodemographic and clinical variables.

Given the non-normal distribution of most variables, non-parametric tests were used. These included the Chi-square test for categorical variables, Spearman’s rank correlation coefficient, Kruskal–Wallis H test for group comparisons, and Mann–Whitney U test for pairwise post hoc analyses.

Latent profile analysis (LPA) was performed to identify homogeneous sensory processing subgroups based on standardized SPM-P domain scores. Model selection was guided by the Bayesian Information Criterion (BIC), entropy values, class size distribution, and the Bootstrap Likelihood Ratio Test (BLRT). A multinomial logistic regression model was applied to identify predictors of latent profile membership.

The significance level was set at *p* < 0.05. Bonferroni correction was applied where appropriate to adjust for multiple comparisons and reduce the risk for type I error.

## 3. Results

### 3.1. Comparisons Between Groups

Significant group difference emerged in cognitive/global developmental functioning (χ^2^_(2)_ = 89.030, *p* < 0.001). Post hoc comparisons revealed that the children in the LD group scored significantly higher than those in the ONDD (*p* < 0.001) and ASD (*p* < 0.001) groups. Although a nominally significant difference was observed between the ONDD and ASD groups (*p* = 0.006), this did not survive correction for multiple comparisons.

For cognitive and global developmental measures, higher scores reflect better functioning. Conversely, for the SPM-P and CBCL 1½–5, higher T-scores indicate greater levels of difficulty or more severe dysfunction.

Regarding parent-reported sensory processing profiles, as assessed by the SPM-P, significant group differences emerged across several domains. Specifically, statistically significant differences were found in social participation (χ^2^_(2)_ = 38.07, *p* < 0.001), vision (χ^2^_(2)_ = 14.84, *p* < 0.001), touch (χ^2^_(2)_ = 13.52, *p* = 0.001), taste and smell (χ^2^_(2)_ = 20.47, *p* < 0.001), balance and motion (χ^2^_(2)_ = 16.46, *p* < 0.001), planning and ideas (χ^2^_(2)_ = 25.40, *p* < 0.001), and in the SPM-P total score (χ^2^_(2)_ = 18.42, *p* < 0.001). No significant differences were found in the hearing (χ^2^_(2)_ = 6.37, *p* = 0.041) and body awareness (χ^2^_(2)_ = 9.78, *p* = 0.008) domains after correction.

Post hoc analyses revealed that the LD group scored significantly lower than both the ONDD and ASD groups in social participation (*p* = 0.002 and *p* < 0.001, respectively) and planning and ideas (*p* = 0.015 and *p* < 0.001, respectively); significant differences between ONDD and ASD groups were also observed for these two domains (*p* = 0.025 and *p* = 0.023, respectively). In the vision and taste and smell domains, the LD group again showed significantly lower scores compared to both the ONDD (*p* = 0.004 and *p* = 0.014, respectively) and ASD (*p* = 0.001 and *p* < 0.001, respectively) groups, while no significant differences were found between the ONDD and ASD groups. Regarding touch and balance and motion domains, the LD group scored significantly lower than the ASD group (*p* = 0.010 and *p* < 0.010, respectively), whereas comparisons between the LD and ONDD groups and between the ONDD and ASD groups did not reach significance. Finally, the SPM-P total score was significantly lower in the LD group compared to both the ONDD (*p* = 0.011) and ASD (*p* < 0.001) groups, with no significant differences between ONDD and ASD. Detailed pairwise comparisons are reported in Table 2.

As for parent ratings of children’s emotional and behavioral functioning, assessed through the CBCL 1½-5, significant group differences were found in the Total Problems score (χ^2^_(2)_ = 11.75, *p* = 0.003) and in the Internalizing Problems composite scale (χ^2^_(2)_ = 21.40, *p* < 0.001). Additional significant differences emerged in several DSM-oriented scales, including Affective Problems (χ^2^_(2)_ = 12.20, *p* = 0.002), Pervasive Developmental Problems (χ^2^_(2)_ = 29.62, *p* < 0.001), and Oppositional Defiant Problems (χ^2^_(2)_ = 12.62, *p* = 0.002). No significant differences were found in Externalizing Problems (χ^2^_(2)_ = 8.87, *p* = 0.012), Anxiety Problems (χ^2^_(2)_ = 8.51, *p* = 0.014), and Attention Deficit/Hyperactivity Problems (χ^2^_(2)_ = 9.02, *p* = 0.011), after correction for multiple comparisons.

Post hoc comparisons indicated that the ASD group scored significantly higher than the LD group on Affective Problems (*p* = 0.003), Pervasive Developmental Problems (*p* < 0.001), Attention Deficit/Hyperactivity Problems (*p* = 0.008), Oppositional Defiant Problems (*p* = 0.002), Internalizing Problems (*p* < 0.001), and Total Problems (*p* = 0.003) scales. A significant difference between the LD and ONDD groups emerged only for Internalizing Problems (*p* = 0.037) while a significant difference between the ONDD and ASD groups was found only for Pervasive Developmental Problems (*p* = 0.001). Full pairwise results are provided in Table 3.

### 3.2. Sensory Processing Subgroup Classification Based on LPA

We compared four different latent profile analysis (LPA) models testing the fit of 2–5 latent classes of sensory processing typologies, based on parent-reported scores across the core domains of the SPM-P (social participation, vision, hearing, touch, taste and smell, body awareness, balance and motion, planning and ideas). This analysis aimed to identify subgroups of children who shared similar sensory processing patterns across these domains. Conditional latent profiles were initially estimated without covariates, using only standardized sensory domain scores. Model selection was guided by fit indices including the Bayesian Information Criterion (BIC), entropy values, class size distributions, and the Bootstrap Likelihood Ratio Test (BLRT). BIC assesses overall model fit (with lower values indicating better fit), entropy indicates classification accuracy (values closer to 1 reflect clearer group distinctions), and class size distribution ensures that each group includes a meaningful portion of the sample. The BLRT helps determine whether a model with more profiles fits the data significantly better than a simpler model with fewer profiles.

The three-profile solution yielded the most balanced combination of model fit (BIC = 506.852) and classification accuracy (entropy = 0.879), with well-proportioned and interpretable class sizes (ranging from 20.15% to 44.87%). Although the five-profile model yielded the lowest BIC (506.202), the difference from the three-profile solution was minimal (ΔBIC = 0.65), and entropy was slightly lower (0.869). According to established criteria, such a difference does not provide strong support for model improvement (ΔBIC < 2 indicates weak evidence) [34]. Moreover, the five-profile model produced smaller and less balanced class sizes (ranging from 10.27% to 25.48%). Therefore, the three-profile model was retained as the optimal and most parsimonious model to describe sensory processing subgroups in the sample (see Table 4).

Profile 1—Multisystemic Sensory Dysfunction (MSD)

The first identified profile included 20.1% of the sample (*n* = 53) and was characterized by elevated scores across all sensory domains.

Profile 2—Typical Sensory Processing (TSP)

This was the largest group, accounting for 44.9% of the sample (*n* = 118), and showed the lowest scores across all sensory domains.

Profile 3—Mixed Subclinical Sensory Processing (MSSP)

**Figure 1:**
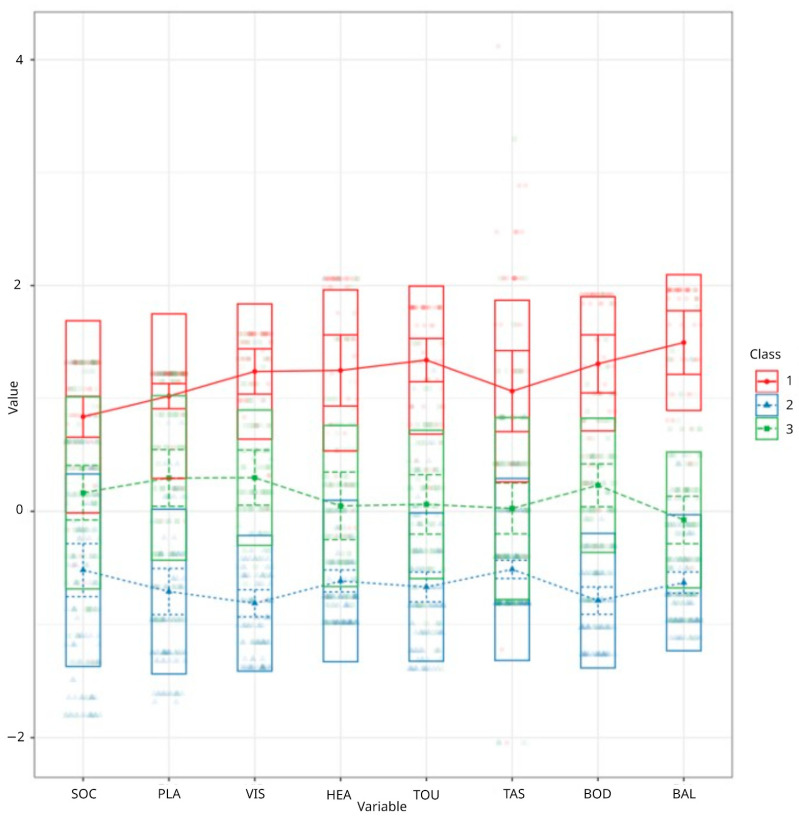
Visualization of three latent sensory profiles. SOC = social participation; PLA = planning and ideas; VIS = vision; HEA = hearing; TOU = touch; TAS = taste and smell; BOD = body awareness; BAL = balance and motion.

The third profile comprised 35% of the sample (*n* = 92). Children in this group exhibited elevated scores specifically in the domains of vision, body awareness, and planning and ideas, while scores across other sensory modalities remained within normative or subthreshold ranges. The three latent sensory profiles identified though LPA are depicted in Figure 1.

### 3.3. Clinical Characterization of Latent Sensory Profiles

An analysis of the population characteristics associated with the identified sensory profiles revealed a significant difference in diagnostic distribution across classes (χ^2^_(4)_ = 27.2, *p* < 0.001). Standardized residual analysis—where values exceeding ±1.96 are considered significantly different from expected (*p* < 0.05)—highlighted that children with LD were significantly overrepresented in the TSP profile (residual = 2654) and underrepresented in the MSD profile (residual = −2530). Conversely, the MSD profile was significantly more frequent in the ASD group (residuals = 2657). The diagnostic breakdown by profile is presented in Table 5.

Subsequent analyses examined emotional, behavioral and sensory processing functioning to determine whether the latent profiles identified through LPA reflected clinically relevant impairments in these domains. Specifically, we aimed to identify areas of greater impairment as well as domains of preserved or typical functioning. Chi-square residual analyses of the distribution of clinical, subclinical, and typical scores revealed significant differences across profiles (χ^2^_(4)_ = 31.9–308, *p* < 0.001). The MSD profile was associated with a significantly higher frequency of clinical scores across all CBCL 1½-5 and SPM-P scales (CBCL 1½-5 Total Problems: residuals = 7.115; SPM-P total score: residuals = 11.790), and a significantly lower frequency of typical scores (CBCL 1½-5 Total Problems: residuals = −4.919; SPM-P total score: residuals = −5.749).

Conversely, the TSP was characterized by significantly fewer clinical scores (CBCL 1½-5 Total Problems: residuals = −4.505; SPM-P total score: residuals = −5.099) and a higher frequency of typical scores across several CBCL 1½–5 and SPM-P scales (CBCL 1½-5 total score: residuals = 3.765; SPM-P total score: residuals = 5.175).

The MSSP profile exhibited a more heterogeneous pattern, with a relatively balanced distribution of clinical, subclinical and typical scores. No significant residuals emerged for most CBCL 1½-5 subscales, except for the Internalizing Problems subscale, where subclinical scores were overrepresented (residuals = 2.820). Regarding the SPM-P, this profile was predominantly characterized by subclinical-range scores across multiple sensory domains (SPM-P total score: residuals = 6.796). Notably, the social participation subscale showed a roughly equal distribution across score categories, while the planning and ideas subscale and the balance and movement subscale showed, respectively, a lower frequency of typical scores (residuals = −2.315) and a lower frequency of clinical scores (residuals = −2.390). Supplementary Tables S1 and S2 report the detailed distribution of clinical, subclinical, and typical scores across CBCL 1½–5 and SPM-P domains for each latent profile identified through LPA.

### 3.4. Predictors of Profile Membership (Multinomial Regression)

Multinomial logistic regression was conducted to investigate whether age, cognitive/developmental functioning, and emotional–behavioral characteristics predicted the membership in the three latent profiles identified through LPA. The MSD profile was used as the reference category. The likelihood ratio test revealed that only two of the nine covariates included significantly contributed to the model: cognitive/developmental functioning (χ^2^_(2)_ = 7.779, *p* = 0.020) and CBCL 1½-5 Internalizing Problems (χ^2^_(2)_ = 8.591, *p* = 0.014).

Compared to the MSD profile, membership in the TSP profile was significantly associated with higher cognitive/developmental functioning (β = 0.022, SE = 0.010, *p* = 0.019, OR = 1.023, 95% CI [1.004, 1.042]) and lower scores on CBCL 1½-5 Internalizing Problems (β = −0.168, SE = 0.070, *p* = 0.016, OR = 0.845, 95% CI [0.737, 0.969]).

In the comparison between the MSSP and the MSD profiles, only the CBCL 1½-5 Anxiety Problems subscale emerged as a significant predictor. Specifically, lower anxiety scores were associated with a greater likelihood of belonging to the MSSP profile (β = −0.070, SE = 0.033, *p* = 0.037, OR = 0.933, 95% CI [0.874, 0.996]).

### 3.5. Emotional–Behavioral Differences Across Profiles

Significant differences among the three profiles identified by LPA emerged across all CBCL 1½–5 scales, as confirmed by non-parametric one-way ANOVA (Kruskal–Wallis). Group effects were statistically significant in all DSM-oriented and composite scales (*p* < 0.001), with effect sizes ranging from moderate to large (ε^2^ = 0.156–0.443). The largest effects were observed for Internalizing Problems (χ^2^_(2)_ = 116.1, ε^2^ = 0.443), Pervasive Developmental Problems (χ^2^_(2)_ = 97.7, ε^2^ = 0.373), Anxiety Problems (χ^2^_(2)_ = 78.2, ε^2^ = 0.298), and Affective Problems (χ^2^_(2)_ = 76.4, ε^2^ = 0.292), followed by Externalizing Problems (χ^2^_(2)_ = 65.1, ε^2^ = 0.249), ADHD (χ^2^_(2)_ = 50.8, ε^2^ = 0.194), and Oppositional Defiant Problems (χ^2^_(2)_ = 40.9, ε^2^ = 0.156).

Post hoc analyses further clarified the pattern of differences, revealing a consistent gradient of symptom severity across the three profiles (see Table 6). Children in the MSD profile consistently exhibited the highest scores across all CBCL 1½–5 scales, significantly exceeding both the MSSP and TSP profiles (*p* < 0.001 for all pairwise comparisons). Additionally, the MSSP profile scored significantly higher than the TSP profile across all scales. Detailed results of pairwise comparisons across CBCL 1½–5 scales are reported in Table 6.

## 4. Discussion

The findings of this study support the growing view that sensory processing atypicalities represent a core, transdiagnostic feature of neurodevelopmental disorders, with meaningful associations with emotional regulation and clinical severity.

### 4.1. Sensory Processing in Neurodevelopmental Conditions: A Transdiagnostic Perspective

Over the past decade, sensory processing abnormalities have received increasing attention as salient features across multiple neurodevelopmental disorders. Although sensory issues have long been recognized as hallmark features of ASD, accumulating evidence suggests that such difficulties are not confined to autism but occur in a range of clinical populations, including children with LD, ADHD, and intellectual disabilities [3,35,36]. The current study contributes to this literature by demonstrating that sensory processing abnormalities are not equally distributed across diagnostic groups and exhibit distinct patterns of severity and domain-specific impairment.

Children with ASD showed the most pervasive sensory dysfunction, with significantly higher scores across all SPM-P domains compared to the LD group. The ONDD group also exhibited elevated sensory scores relative to the LD group, though to a lesser extent than the ASD group. These findings are consistent with dimensional models of neurodevelopment, suggesting that sensory modulation difficulties may reflect shared neurobiological vulnerabilities that cut across categorical diagnoses [37,38].

Interestingly, hearing and body awareness did not show significant differences between groups after correction for multiple comparisons, suggesting that certain sensory modalities may be more diagnostically discriminative than others. Domains such as vision, planning and ideas, and taste and smell emerged as particularly salient in distinguishing LD from ASD and ONDD, consistent with recent findings linking visuo-motor integration and praxis impairments to more severe neurodevelopmental phenotypes [2,39].

### 4.2. Emotional and Behavioral Functioning in Preschool NDDs

Consistent with previous findings, our results indicate that children with ASD exhibit significantly greater emotional and behavioral difficulties compared to their peers with LD and ONDD [40,41]. Notably, the ASD group exhibited significantly higher scores on the Internalizing Problems and Pervasive Developmental Problems scales, as well as on affective and attention-related problems. These findings support the view that co-occurring emotional difficulties are prevalent and often underrecognized in young children with ASD, even at the preschool stage. Emotional and behavioral challenges may not only exacerbate the functional burden experienced by children with ASD but may also complicate early differential diagnosis and access to targeted interventions [42].

Children in the ONDD group showed intermediate profiles of emotional functioning, further supporting the notion of a clinical continuum. While they did not differ significantly from the LD group on most CBCL scales, a higher rate of internalizing difficulties was observed, highlighting the emotional vulnerability of this diagnostically heterogeneous group. Recent findings suggest that children with NDDs often display patterns of emotional dysregulation that do not align neatly with existing categorical diagnoses [43].

These results highlight the co-occurrence of emotional and behavioral symptoms across the NDD sample, with particularly high levels of impairment observed in children with ASD. These vulnerabilities contribute to a more compromised clinical profile and may negatively affect overall quality of life [44,45,46]. Notably, children with mixed or nonspecific diagnoses (ONDD) also showed subclinical sensory difficulties alongside elevated internalizing symptoms. This pattern underscores the need for early screening protocols that assess both sensory and emotional domains—even in children without a specific ASD or LD diagnosis. Identifying such vulnerabilities in preschoolers may help prevent the escalation of symptoms and support more adaptive emotional, academic, and social outcomes later in development [47,48].

### 4.3. Latent Sensory Profiles: From Categorization to Dimensionality

The identification of three latent sensory profiles through LPA enriches our understanding of individual variability in sensory processing beyond categorical diagnoses. The MSD profile, characterized by globally elevated sensory scores (e.g., social participation, vision, hearing, touch, taste/smell, body awareness, balance and motion, and planning and ideas domains), is consistent with a globally dysregulated sensory processing pattern, indicative of pervasive deficits in both sensory modulation and higher-order integrative functions. It encompassed 20% of the sample and was significantly overrepresented in children with ASD. This profile aligns with previous findings linking multisensory modulation difficulties to more severe neurodevelopmental phenotypes and broader impairments in social and adaptive functioning [2,6].

In contrast, the TSP profile—comprising nearly 45% of the sample—consistently obtained the lowest scores across all sensory domains, reflecting a globally modulated and developmentally normative sensory profile. Children within this group displayed minimal signs of sensory-related challenges or difficulties in higher-order sensory integration. The profile was predominantly composed of children with LD and was associated with low levels of sensory and emotional dysfunction. This pattern highlights the relative preservation of regulatory functions in this subgroup and reinforces the heterogeneity observed across neurodevelopmental conditions. These findings are consistent with previous studies showing that not all children with neurodevelopmental disorders exhibit clinically significant sensory symptoms [2,49]. Importantly, they caution against overgeneralizations in clinical practice and underscore the value of individualized sensory and behavioral assessments.

The MSSP profile represents an intermediate cluster, marked by subclinical-range scores across multiple sensory domains. Children in this group exhibited elevated scores specifically in the domains of vision, body awareness, and planning and ideas, while scores across other sensory modalities remained within normative or subthreshold ranges. This pattern suggests a circumscribed sensory processing profile, characterized by selective difficulties in visuo-motor integration and ideational praxis, in the absence of generalized sensory modulation deficits. The presence of this profile in a substantial proportion of the sample (35%) suggests that subclinical sensory difficulties may be common across NDDs and could represent early markers of later-emerging regulatory challenges. This finding aligns with dimensional models of neurodevelopment, which conceptualize subthreshold traits as clinically relevant and predictive of functional outcomes across time [2,43,50].

Furthermore, the need for refined sensory assessment approaches is underscored by recent systematic reviews, which highlight inconsistencies in existing caregiver- and self-report measures and their limited capacity to capture the complexity of multidomain sensory profiles frequently observed in heterogeneous neurodevelopmental populations [51].

### 4.4. Sensory–Emotional Coupling: A Developmental System Model

The robust association between sensory profiles and emotional–behavioral functioning observed in this study lends support to developmental system models of psychopathology. According to these models, early sensory modulation capacity influences a child’s ability to engage with the environment, regulate affect, and acquire adaptive strategies [52]. Children within the MSD profile exhibited the highest levels of emotional and behavioral dysregulation, including elevated internalizing and externalizing symptoms. Conversely, the TSP profile was associated with normative emotional functioning, while the MSSP profile demonstrated subclinical emotional vulnerability. These findings are consistent with longitudinal evidence showing that early sensory processing patterns can predict later adaptive, maladaptive, and participation outcomes in both autistic and non-autistic children [53]. These findings also resonate with theories of sensory–emotional integration, which propose that patterns such as sensory over-responsivity and under-responsivity are linked to emotion regulation difficulties, anxiety, and mood dysregulation [19]. Recent work has further demonstrated that specific sensory dimensions—such as tactile hypersensitivity or poor interoceptive awareness—may be uniquely associated with distinct profiles of affective dysregulation in NDDs [54,55].

The fact that sensory profiles were associated with emotional–behavioral outcomes—even when controlling for diagnosis—highlights the transdiagnostic utility of incorporating sensory processing assessments into early clinical evaluation. This approach may also help identify children at risk for emotional comorbidities before such symptoms become clinically overt.

### 4.5. Predictive Value of Cognitive and Emotional Variables

Our regression analyses revealed that cognitive functioning and internalizing symptoms significantly predicted profile membership. Higher cognitive scores and fewer internalizing symptoms were associated with increased odds of belonging to the TSP profile, whereas lower anxiety scores predicted membership in the MSSP profile over the MSD group. These findings suggest that sensory dysregulation may index broader neurodevelopmental compromise, encompassing both cognitive and affective domains.

These results are consistent with prior evidence showing that children with greater emotion regulation skills exhibit reduced functional impairment in the context of sensory over-responsivity. Emotion regulation may act as a protective factor, mitigating the adverse effects of sensory difficulties on behavioral and social outcomes, independently of diagnostic status [56,57].

Importantly, these predictors were significant independently of diagnostic group, reinforcing the notion that neurodevelopmental risk is better conceptualized as a dynamic interplay of traits rather than a fixed diagnostic label. This view is also supported by recent initiatives promoting a dimensional framework for understanding child psychopathology, such as the Hierarchical Taxonomy of Psychopathology (HiTOP) and Research Domain Criteria (RDoC), which emphasize the integration of biological, cognitive, and behavioral domains [21,58]. Sensory functioning may represent a key entry point in such frameworks, particularly in early developmental stages.

## 5. Conclusions

The present study provides novel insights into the sensory–emotional heterogeneity of preschool-aged children with neurodevelopmental disorders (NDDs), using a person-centered approach. Unlike prior research focusing on categorical comparisons, we employed latent profile analysis (LPA) to identify transdiagnostic sensory subtypes across three clinical groups: autism spectrum disorder (ASD), language disorder (LD), and other neurodevelopmental disorders (ONDD). This methodology allowed us to uncover data-driven sensory profiles that reflect varying levels of impairment and emotional vulnerability, independent of specific diagnostic labels.

A key contribution of this study is the identification of a distinct subclinical sensory–emotional phenotype—marked by selective difficulties in visuo-motor integration and ideational praxis—which was most frequently observed in the ONDD group. This finding challenges a strictly diagnosis-based framework and highlights the relevance of dimensional assessment strategies in early childhood.

Moreover, the integration of sensory, emotional–behavioral, and cognitive data within a single analytic model offers a more ecologically valid understanding of functional profiles in NDDs. Our results revealed that altered sensory processing was closely associated with internalizing symptoms, particularly in children with ASD, but also in those with nonspecific developmental concerns. Regression analyses further indicated that higher cognitive functioning and lower emotional dysregulation predicted more normative sensory profiles.

These findings underscore the developmental relevance of sensory processing across diagnostic categories and support the inclusion of sensory profiling in early evaluation protocols. The results also revealed a clear association between altered sensory processing and emotional dysregulation, particularly in children with autism spectrum disorder. Although sensory features are sometimes under-recognized compared to more overt symptoms, our data suggest they warrant systematic attention in developmental assessments to support more adaptive trajectories. Identifying specific sensory–emotional phenotypes may enhance the precision of early interventions and help address emotional vulnerabilities before they consolidate into more severe psychopathology. In addition, promoting more inclusive environments that reduce sensory overload and related distress may contribute to improvements in children’s emotional and behavioral functioning.

## 6. Limitations

Although this study contributes to the transdiagnostic understanding of sensory processing in early neurodevelopment, several limitations should be acknowledged.

First, the diagnostic groups were imbalanced in size, with an overrepresentation of children with ASD relative to those with LD and ONDD. Future research should aim to include more balanced samples and consider increasing female representation to improve the generalizability of findings.

Second, the study relied primarily on parent-report measures for sensory and behavioral functioning. Although these instruments offer ecological validity and practical value in clinical settings, they may be subject to reporting biases and parental perceptions. Incorporating clinician-rated or performance-based sensory assessments would strengthen the robustness of future findings.

Third, although the three-profile solution was parsimonious and clinically meaningful in our preschool sample, previous studies in older children using different instruments identified a five-profile structure [2]. These discrepancies highlight the influence of developmental stage, sample characteristics, and assessment tools on latent class modeling. Future research should employ multimodal, age-sensitive sensory assessments to better capture the complexity and variability of sensory processing across neurodevelopmental trajectories.

## Figures and Tables

**Table 1:** Demographic characteristics of the sample by diagnostic group.

	Total Sample (*n* = 263)	LD Group (*n* = 73)	ONDD Group (*n* = 75)	ASD Group (*n* = 115)
Males (%)	213 (80.99%)	58 (79.45%)	62 (82.67%)	93 (80.87%)
Females (%)	50 (19.01%)	15 (20.55%)	13 (17.33%)	22 (19.13%)
Age in months, mean (SD)	43.36 (11.34)	44.49 (10.77)	43.63 (11.27)	42.47 (11.76)

LD = language disorder; ONDD = other neurodevelopmental disorder; ASD = autism spectrum disorder; SD = standard deviation.

**Table 2:** Between-group comparisons of the Sensory Processing Measure—Preschool.

SPM-P Scores, Mean (SD)	Total Sample (*n* = 263)	LD Group (*n* = 73)	ONDD Group (*n* = 75)	ASD Group (*n* = 115)	Post Hoc	*p*-Value
SOC, T-score	63.1 (12.8)	55.6 (12.8)	63 (12.5)	67.9 (10.7)	LD vs. ONDD	0.002 *
					LD vs. ASD	<0.001 *
					ONDD vs. ASD	0.025 *
VIS, T-score	58.7 (13.6)	53.5 (12.1)	60.4 (13.3)	60.9 (13.8)	LD vs. ONDD	0.004 *
					LD vs. ASD	0.001 *
					ONDD vs. ASD	0.987
TOU, T-score	57.4 (12.5)	53.2 (10.4)	56.5 (11.2)	60.6 (13.7)	LD vs. ONDD	0.175
					LD vs. ASD	0.001 *
					ONDD vs. ASD	0.137
TAS, raw score	5.98 (2.43)	5.03 (2.16)	6.32 (2.29)	7.02 (3.09)	LD vs. ONDD	0.014 *
					LD vs. ASD	<0.001 *
					ONDD vs. ASD	0.260
BAL, T-score	54.5 (13)	49.8 (10.3)	53.7 (13)	58.1 (13.7)	LD vs. ONDD	0.332
					LD vs. ASD	<0.001 *
					ONDD vs. ASD	0.056
PLA, T-score	63.2 (13.8)	57 (12.7)	62.6 (12.4)	67.6 (13.8)	LD vs. ONDD	0.015 *
					LD vs. ASD	<0.001 *
					ONDD vs. ASD	0.023 *
TOT, T-score	57.6 (13)	52.3 (10.4)	57.5 (11.9)	61.1 (14.2)	LD vs. ONDD	0.011 *
					LD vs. ASD	<0.001 *
					ONDD vs. ASD	0.061

SPM-P = Sensory Processing Measure—Preschool; SD = standard deviation; LD = language disorder; ONDD = other neurodevelopmental disorder; ASD = autism spectrum disorder; SOC = social participation; VIS = vision; TOU = touch; TAS = taste and smell; BAL = balance and motion; PLA = planning and ideas; TOT = total sensory systems score. * *p* < 0.05.

**Table 3:** Between-group comparisons of the Child Behavior Checklist for Ages 1½-5 years.

CBCL 1½-5 T-Scores, Mean (SD)	Total Sample (*n* = 263)	LD Group (*n* = 73)	ONDD Group (*n* = 75)	ASD Group (*n* = 115)	Post Hoc	*p*-Value
DSM-oriented scales						
Affective Problems	57.2 (7.91)	55.3 (7.04)	56.6 (8.27)	58.6 (8.20)	LD vs. ONDD	0.181
					LD vs. ASD	0.003 *
					ONDD vs. ASD	0.138
Pervasive Developmental Problems	65.1 (10.8)	60.6 (9.42)	63.5 (10.14)	69 (10.9)	LD vs. ONDD	0.145
					LD vs. ASD	<0.001 *
					ONDD vs. ASD	0.001 *
Oppositional Defiant Problems	54.8 (6.64)	53.5 (6.21)	54.3 (6.39)	56 (7.14)	LD vs. ONDD	0.201
					LD vs. ASD	0.002 *
					ONDD vs. ASD	0.161
Composite scales						
Internalizing	57.2 (11.5)	52.3 (11.7)	56.8 (10.6)	60.6 (10.9)	LD vs. ONDD	0.037 *
					LD vs. ASD	<0.001 *
					ONDD vs. ASD	0.055
Total	57.1 (11.5)	53.5 (10.5)	56.9 (10.6)	59.5 (12.2)	LD vs. ONDD	0.141
					LD vs. ASD	0.003 *
					ONDD vs. ASD	0.231

CBCL 1½-5 = Child Behavior Checklist for Ages 1½-5; SD = standard deviation; LD = language disorder; ONDD = other neurodevelopmental disorder; ASD = autism spectrum disorder; * *p* < 0.05.

**Table 4:** Latent profile analysis model selection.

Number of Classes	BIC	Entropy	Smallest Class (%)	Largest Class (%)
2	522,878.74	0.936	28.90%	71.10%
**3**	**506,852.38**	**0.879**	**20.15%**	**44.87%**
4	507,186.63	0.807	19.39%	28.90%
5	506,202.14	0.869	10.27%	25.48%

BIC = Bayesian Information Criterion.

**Table 5:** Diagnostic distribution across sensory processing profiles.

Sensory Profiles	Diagnosis	Observed (O)	Expected (E)	Std. Residuals (RS)
MSD	LD	5	14.7	−2.53 *
	ONDD	12	15.1	−0.79
	ASD	36	23.2	2.66 *
TSP	LD	48	32.8	2.65 *
	ONDD	30	33.7	−0.64
	ASD	40	51.6	1.61
MSSP	LD	20	25.5	−1.09
	ONDD	33	26.2	1.33
	ASD	39	40.2	−0.19

MSD = Multisystemic Sensory Dysfunction; TSP = Typical Sensory Processing; MSSP = Mixed Subclinical Sensory Processing; LD = language disorder; ONDD = other neurodevelopmental disorder; ASD = autism spectrum disorder. * *p* < 0.05.

**Table 6:** Post hoc comparisons of CBCL 1½–5 DSM-oriented and composite scale scores across latent profiles.

CBCL 1½-5 T-Scores, Mean (SD)	MSD (*n* = 53)	TSP (*n* = 118)	MSSP (*n* = 92)	Post Hoc	*p*-Value
DSM-oriented scales					
Affective Problems	64.6 (8.8)	53.5 (4.8)	57.6 (7.5)	MSD vs. TSP	<0.001 *
				MSD vs. MSSP	<0.001 *
				TSP vs. MSSP	<0.001 *
Anxiety Problems	67.3 (10.3)	53.2 (5.6)	56.4 (7.1)	MSD vs. TSP	<0.001 *
				MSD vs. MSSP	<0.001 *
				TSP vs. MSSP	<0.001 *
Pervasive Developmental Problems	76.7 (8.3)	59.3 (8.4)	65.8 (9.1)	MSD vs. TSP	<0.001 *
				MSD vs. MSSP	<0.001 *
				TSP vs. MSSP	<0.001 *
Attention Deficit/Hyperactivity Problems	64.4 (7.6)	55.1 (5.8)	58 (7.2)	MSD vs. TSP	<0.001 *
				MSD vs. MSSP	<0.001 *
				TSP vs. MSSP	0.007 *
Oppositional Defiant Problems	60 (8.1)	52.7 (4.7)	54.5 (6.3)	MSD vs. TSP	<0.001 *
				MSD vs. MSSP	<0.001 *
				TSP vs. MSSP	0.015 *
Composite scales					
Internalizing	69.6 (7.5)	50.1 (9.8)	59.2 (8.2)	MSD vs. TSP	<0.001 *
				MSD vs. MSSP	<0.001 *
				TSP vs. MSSP	<0.001 *
Externalizing	64.4 (11.4)	50.1 (4.1)	55.8 (9.4)	MSD vs. TSP	<0.001 *
				MSD vs. MSSP	<0.001 *
				TSP vs. MSSP	<0.001 *
Total	70.5 (9.4)	50.1 (8.5)	58.3 (8.4)	MSD vs. TSP	<0.001 *
				MSD vs. MSSP	<0.001 *
				TSP vs. MSSP	<0.001 *

CBCL 1½-5 = Child Behavior Checklist for Ages 1½-5; SD = standard deviation; MSD = Multisystemic Sensory Dysfunction; TSP = Typical Sensory Processing; MSSP = Mixed Subclinical Sensory Processing. * *p* < 0.05.

## Data Availability

Data supporting the findings of this study are available within the article. Further information can be requested from the corresponding authors. Due to privacy considerations, additional data are not publicly available.

## Supplementary Materials

The following supporting information can be downloaded at https://www.mdpi.com/article/10.3390/children12070958/s1: Table S1: Distribution of CBCL 1½-5 scores in latent sensory profiles; Table S2: Distribution of SPM-P scores in latent sensory profiles.
