# Supplementary material for: Feeling the World Differently: Sensory and Emotional Profiles in Preschool Neurodevelopmental Disorders
Source: Children (Basel). 2025 Jul 21;12(7):958. doi: 10.3390/children12070958 (PMC12293204; doi:10.3390/children12070958)
Supplement: Supplementary file 1 [file children-12-00958-s001.zip › children-3735630-supplementary.pdf]

Table S1. Distribution of CBCL 1½-5 Scores in Latent Sensory Profiles

DSM-oriented scales	Sensory Profiles		Observed (O)	Expected (E)	Std. Residuals (RS)
Affective problems	MSD	Normal	31	45.34	-2.13*
		Borderline	6	1.81	3.11*
		Clinical	16	5.84	4.20*
	TSP	Normal	114	100.95	1.30
		Borderline	0	4.04	-2.01*
		Clinical	4	13.01	-2.50*
	MSSP	Normal	80	78.71	0.14
		Borderline	3	3.15	-0.08
		Clinical	9	10.14	-0.36
Anxiety problems	MSD	Normal	21	42.72	-3.32*
		Borderline	10	3.83	3.15*
		Clinical	22	6.45	6.12*
	TSP	Normal	111	95.12	1.63
		Borderline	5	8.52	-1.20
		Clinical	2	14.36	-3.26*
	MSSP	Normal	80	74.16	0.68
		Borderline	4	6.65	-1.03
		Clinical	8	11.19	-0.95
Pervasive developmental problems	MSD	Normal	4	25.39	-4.24*
		Borderline	3	7.46	-1.63
		Clinical	46	20.15	5.76*
	TSP	Normal	83	56.5	3.52*
		Borderline	17	16.6	0.09
		Clinical	18	44.9	-4.01*
	MSSP	Normal	39	44.1	-0.77
		Borderline	17	12.9	1.14
		Clinical	36	35	0.17
Attention deficit/hyperactivity problems	MSD	Normal	32	43.13	-1.69
		Borderline	5	4.23	0.37
		Clinical	16	5.64	4.36*
	TSP	Normal	108	96.02	1.22
		Borderline	6	9.42	-1.11
		Clinical	4	12.56	-2.41*
	MSSP	Normal	74	74.86	-0.09
		Borderline	10	7.35	0.98
		Clinical	8	9.79	-0.57
Oppositional defiant problems	MSD	Normal	32	44.74	-1.90
		Borderline	10	5.04	2.21*
		Clinical	11	3.22	4.33*
	TSP	Normal	109	99.6	0.94
		Borderline	8	11.22	-0.96

		Clinical	1	7.18	-2.31*
	MSSP	Normal	81	77.66	0.38
		Borderline	7	8.75	-0.59
		Clinical	4	5.6	-0.67
Composite scales					
Internalizing	MSD	Normal	5	29.02	-4.46*
		Borderline	5	7.86	-1.02
		Clinical	43	16.12	6.69*
	TSP	Normal	98	64.6	4.15*
		Borderline	10	17.5	-1.79
		Clinical	10	35.9	-4.32*
	MSSP	Normal	41	50.4	-1.32
		Borderline	24	13.6	2.82*
		Clinical	27	28	-0.19
Externalizing	MSD	Normal	17	37.68	-3.37*
		Borderline	9	5.24	1.64
		Clinical	27	10.08	5.33*
	TSP	Normal	103	83.9	2.08*
		Borderline	8	11.7	-1.08
		Clinical	7	22.4	-3.25*
	MSSP	Normal	67	65.41	0.19
		Borderline	9	9.1	-0.03
		Clinical	16	17.49	-0.36
Total	MSD	Normal	5	33.45	-4.92*
		Borderline	6	4.84	0.53
		Clinical	42	14.71	7.11*
	TSP	Normal	107	74.5	3.76*
		Borderline	4	10.8	-2.07*
		Clinical	7	32.8	-4.50*
	MSSP	Normal	54	58.07	-0.53
		Borderline	14	8.4	1.93
		Clinical	24	25.54	-0.30

MSD = Multisystemic Sensory Disfunction; TSP = Typical Sensory Processing; MSSP = Mixed Subclinical Sensory Processing.

Table S2. Distribution of SPM-P Scores in Latent Sensory Profiles

SPM-P	Sensory Profiles		Observed (O)	Expected (E)	Std. Residuals (RS)
SOC	MSD	Normal	4	19.5	-3.51*
		Borderline	8	13.9	-1.58
		Clinical	41	19.5	4.86*
	TSP	Normal	66	43.5	3.41*
		Borderline	31	31	0
		Clinical	21	43.5	-3.41*
	MSSP	Normal	27	33.9	-1.18

		Borderline	30	24.1	1.20
		Clinical	35	33.9	0.18
VIS	MSD	Normal	2	29.62	-5.07*
		Borderline	5	9.47	-1.45
		Clinical	46	13.9	8.61*
	TSP	Normal	111	66	5.54*
		Borderline	6	21.1	-3.29*
		Clinical	1	31	-5.39*
	MSSP	Normal	34	51.4	-2.43*
		Borderline	36	16.4	4.84*
		Clinical	22	24.1	-0,43
HEA	MSD	Normal	13	39.3	-4.19*
		Borderline	8	5.04	1,32
		Clinical	32	8.67	7.92*
	TSP	Normal	115	87.5	2.94*
		Borderline	1	11.2	-3.05*
		Clinical	2	19.3	-3.94*
	MSSP	Normal	67	68.21	-0.15
		Borderline	16	8.75	2.45*
		Clinical	9	15.04	-1.56
TOU	MSD	Normal	2	31	-5.21*
		Borderline	13	11.7	0.38
		Clinical	38	10.3	8.63*
	TSP	Normal	104	69.1	4.19*
		Borderline	11	26	-2.94*
		Clinical	3	22.9	-4.16*
	MSSP	Normal	48	53.9	-0.80
		Borderline	34	20.3	3.04*
		Clinical	10	17.8	-1.85
BOD	MSD	Normal	6	35.06	-4.91*
		Borderline	14	9.67	1.39
		Clinical	33	8.26	8.61*
	TSP	Normal	116	78.1	4.29*
		Borderline	2	21.5	-4.20*
		Clinical	0	18.4	-4.29*
	MSSP	Normal	52	60.9	-1.14
		Borderline	32	16.8	3.71*
		Clinical	8	14.3	-1.66
BAL	MSD	Normal	4	37.28	-5.45*
		Borderline	9	6.05	1.19
		Clinical	40	9.67	9.75*
	TSP	Normal	112	83	3.18*
		Borderline	5	13.5	-2.31*
		Clinical	1	21.5	-4.42*

	MSSP	Normal	69	64.7	0.53
		Borderline	16	10.5	1.69
		Clinical	7	16.8	-2.39*
PLA	MSD	Normal	1	24.18	-4.71*
		Borderline	5	7.86	-1.02
		Clinical	47	20.96	5.69*
	TSP	Normal	92	53.8	5.21*
		Borderline	16	17.5	-0.36
		Clinical	10	46.7	-5.37*
	MSSP	Normal	27	42	-2.31*
		Borderline	18	13.6	1.19
		Clinical	47	36.4	1.76
TOT	MSD	Normal	0	33.05	-5.75*
		Borderline	1	8.26	-2.53*
		Clinical	52	11.69	11.79*
	TSP	Normal	118	73.6	5.17*
		Borderline	0	18.4	-4.29*
		Clinical	0	26	-5.09*
	MSSP	Normal	46	57.4	-1.50
		Borderline	40	14.3	6.79*
		Clinical	6	20.3	-3.17*

SOC = socialization; VIS = vision; HEA = hearing; TOU = touch; BOD = body awareness; BAL = balance and motion; PLA = planning and ideas; TOT = total sensory system score; MSD = Multisystemic Sensory Disfunction; TSP = Typical Sensory Processing; MSSP = Mixed Subclinical Sensory Processing.
